# Cardiovascular function shows early impairment in asymptomatic adolescents diagnosed with type 1 diabetes mellitus: an ultrasound-derived myocardial work study

**DOI:** 10.3389/fcvm.2024.1476456

**Published:** 2025-02-05

**Authors:** Martina Ghirardo, Irene Cattapan, Jolanda Sabatino, Alice Pozza, Jennifer Fumanelli, Martina Avesani, Joaquin Gutierrez De Rubalcava Doblas, Carlo Moretti, Biagio Castaldi, Giovanni Di Salvo

**Affiliations:** ^1^ Division of Pediatric Cardiology, Department of Women’s and Children’s Health, University Hospital of Padua, Padua, Italy; ^2^ Pediatric Department, San Bortolo Hospital, Vicenza, Italy; ^3^ PhD School in Developmental Medicine and Health Planning Sciences, University of Padua, Padua, Italy; ^4^ Division of Cardiology, Department of Medical and Surgical Science, Magna Graecia University, Catanzaro, Italy; ^5^ Pediatric Diabetology, Department of Women’s and Children’s Health, University Hospital of Padua, Padua, Italy

**Keywords:** GLS, myocardial work, type 1 diabetes mellitus, diabetic adolescents, advanced echocardiography

## Abstract

**Background:**

Cardiac dysfunction and endothelial damage are known complications of type 1 diabetes mellitus (T1D) mainly affecting adults. However, some studies have shown that subclinical myocardial impairment already present during adolescence. Myocardial work (MW) has emerged as an afterload-independent tool that allows early identification of subclinical damage. This study aims to provide a comprehensive non-invasive cardiovascular evaluation of T1D adolescents using both conventional and advanced echocardiography.

**Methods:**

We enrolled 31 patients, aged between 13 and 19 years, who were diagnosed with T1D for at least 10 years and were followed up by the Paediatric Diabetology Unit of our institution. We collected data relating to anthropometry, lifestyle, blood tests, glycemic control parameters, and conventional and advanced echocardiographic measurements. A comparison of MW parameters with the data from 31 age- and sex-matched healthy volunteers from a previous study in our lab was carried out.

**Results:**

In our population, the glycemic control parameters showed suboptimal control. While diastolic parameters were in the normal range for all the patients, *E*′ velocities and left atrial diameter were significantly worse in patients with poorer glycemic controls. Global longitudinal strain (GLS), global work index (GWI), and global work efficiency (GWE) were significantly lower in the T1D population compared to those in the healthy population (*p* < 0.001), while global wasted work was significantly higher in the T1D population (*p* < 0.001). Patients with stage 1 hypertension or a pre-hypertensive state exhibited pathological pulse wave velocities with values exceeding 8 m/s (>99th percentile).

**Conclusions:**

To the best of our knowledge, this was the first study to investigate MW in T1D adolescents. The descriptive parameters of GLS and MW showed subclinical cardiac damage already during this timeframe. Therefore, these tools should be integrated into the cardiovascular assessment of diabetic adolescents, and preventive strategies should be implemented to maximize glycemic and pressure control effectiveness.

## 1 Introduction

Cardiovascular risk assessment in diabetic adolescents has become critically important in preventive medicine. Type 1 diabetes mellitus (T1D) is one of the most common endocrine and metabolic conditions in childhood. Echocardiography and vascular function parameters in T1D adolescents with type 1 diabetes mellitus (T1D) are significantly altered. In this age group, research suggests a correlation between T1D and early cardiac health issues (1, 2). From an echocardiographic perspective, conventional parameters often reveal a higher incidence of structural and functional alterations in cardiac chambers, such as left ventricular thickening and compromised diastolic function (3–5). Furthermore, several studies have identified significant reductions in global longitudinal strain (GLS), an indicator of myocardial function, in T1D adolescents compared to their healthy peers. This suggests that diabetes may impact heart contractility even at a young age. These changes may have implications for long-term cardiovascular health. Regarding vascular function, investigations into pulse wave velocity (PWV) have detected increased arterial stiffness in young individuals with T1D (6, 7). This increase may be associated with a higher risk of cardiovascular complications throughout life.

However, all the cardiac parameters studied in this population are dependent on afterload, so it is possible that the abnormal measurements are only a reflection of increased afterload.

Myocardial work (MW), an innovative technique that integrates left ventricular pressure into strain measurement on echocardiography, was officially approved and introduced to the market in 2018. In contrast to GLS, it assesses LV performance by integrating afterload determination via blood pressure cuff, thereby offering a measure that is less dependent on afterload (8).

Therefore, this study aims to provide a comprehensive non-invasive cardiovascular evaluation of T1D adolescents using conventional echocardiography, speckle tracking analysis, and for the first time myocardial work.

## 2 Methods

This cross-sectional single-center study was conducted at the Paediatric Department of the University Hospital of Padua between June 2023 and October 2023. A summary outline of the study design and its main results can be found in Supplementary Figure 1.

### 2.1 Population

The inclusion criteria were patients diagnosed with type 1 diabetes mellitus for at least 10 years with intensive therapeutic management. The exclusion criteria were patients diagnosed with diabetes other than type 1, patients without glycemic reports, patients already diagnosed with cardiovascular or congenital heart diseases, patients with genetic cardiological diseases, and patients with a poor acoustic window potentially affecting data analysis.

Thus, we enrolled 31 patients, aged between 13 and 19 years, who were diagnosed with type 1 diabetes mellitus for at least 10 years and who were followed up by the Paediatric Diabetology Unit of our institution. The diagnosis of diabetes followed the criteria defined by the International Society for Paediatric and Adolescent Diabetes (ISPAD) (9). Our cardiological evaluation was intended as best practice in the follow-up for these patients. Informed consent was obtained directly from patients and their parents.

The GLS and myocardial work parameters were then compared to data from 31 age- and sex-matched healthy volunteers from a previous study in our lab (10). The inclusion criteria were patients aged 13–19 years, patients with no previous history of cardiovascular or lung disease and no abnormalities during physical examination (except for a physiologic heart murmur), and patients with normal ECG and normal standard echocardiography. Subjects with minor defects, such as small atrial septal defect, patent ductus arteriosus, and irregular rhythm, or those with images of poor quality were excluded.

### 2.2 Clinical data

We collected clinical data relating to vital parameters and puberal stage from all patients. The puberal stage was determined according to Tanner's classification.

### 2.3 Glycaemic control parameters

The following indices of glycaemic control were studied:

• glycated hemoglobin during the last consultation, target values <7%;
• time in range (TIR—the amount of monitored time with glycemia with values between 70 and 180 mg/dl), target values >70%. In our study, we considered three categories of time in range: TIR > 70%, TIR between 50% and 70%, and TIR inferior to 50%;
• time above range (TAR—the amount of monitored time with glycemia with values between 180 and 250 mg/dl), target values <25%;
• time very above range (TVAR— the amount of monitored time with glycemia with values > 250 mg/dl), target values <5%; and
• coefficient of variation (CV), optimal glycemic variability ≤36%.

### 2.4 Conventional echocardiography

All patients underwent a standard echocardiographic assessment using a Vivid E95 Ultrasound Machine (GE Vingmed Ultrasound AS, Horten, Norway) equipped with a 4Vc probe. Echocardiograms were performed by one pediatric cardiologist (MG). Images were then analyzed offline on the EchoPAC software version 204 by a single experienced reader (IC) blinded to the clinical status and laboratory data. According to the latest recommendations, the conventional parameters of systolic and diastolic function were measured, namely, left ventricular volume, cardiac mass, LV ejection fraction, left atrial anteroposterior diameter, left atrial volume, atrioventricular valve inflow, and annular velocity. The left ventricular end-diastolic volume was calculated by averaging the end-diastolic volumes measured from the four-chamber and two-chamber views. The cardiac mass was defined using the Devereux formula, starting from interventricular septal thickness, posterior wall thickness, and left ventricular end-diastolic diameter on a parasternal long-axis view. The values were then indexed by both body surface area (BSA) and height elevated to 2.7 (11). The obtained values were then interpreted according to previous studies (12). The ejection fraction was assessed using the biplane Simpson method. The left atrial anteroposterior diameter was measured at the end systole on a 2D parasternal long-axis view, while the left atrial volume was estimated from an apical four-chamber view in the same phase of the cardiac cycle. Mitral inflow velocities were measured by placing the pulsed wave Doppler cursor at the tips of the mitral leaflets on an apical four-chamber view. Peaks E and A were consequently identified. Tissue Doppler imaging (TDI) analysis of diastolic velocities was performed from the same view by placing the pulsed wave Doppler on the medial and lateral sides of the mitral annulus to define septal and lateral *E*′, respectively.

### 2.5 Speckle tracking analysis

In addition, atrial strain and left ventricular longitudinal and circumferential strain (CS) were determined by speckle tracking. To ensure optimal tracking, we obtained images with a frame rate between 50 and 80 fps and a sinus rhythm and heart rate variability ≤10% (13). According to the European Association of Cardiovascular Imaging consensus document, the left ventricular longitudinal strain (GLS) was calculated from three apical views (apical four-chamber, apical two-chamber, and apical three-chamber views) (14). The left ventricle circumferential strain was calculated from a parasternal short-axis view at the level of the papillary muscles. The left atrial strain was assessed from an apical four-chamber view (15). Once the images were collected, the endocardial border was traced. The automatized tracking given by the software was inspected before proceeding to the results: The tracking was considered good when it followed the endocardial border throughout the cardiac cycle. We reported peak systolic strain for the GLS and CS, while for atrial strain, we considered the positive deformation occurring during the reservoir phase.

### 2.6 Myocardial work analysis

Once the strain analysis curves from the three apical views were obtained, a dedicated function of the GE software was used to estimate myocardial work (MW). Systolic and diastolic blood pressure values, obtained non-invasively using a digital sphygmomanometer with brachial cuff, were entered into the software. The time of aortic and mitral valve opening and closing was identified by the operator based on the three-chamber recording, as required for the synchronization of strain and pressure data (10).

Therefore, the global work index (GWI, mmHg%) was calculated, together with the derived parameters:

• global constructive work (GCW, mmHg%): work performed by a segment during shortening in systole plus negative work during lengthening in isovolumetric relaxation;
• global wasted work (GWW, mmHg%): negative work performed by one segment during lengthening in systole plus work performed during shortening in isovolumic relaxation; and
• global work efficiency (GWE): constructive work divided by the sum of constructive work and wasted work (0%–100%).

### 2.7 Pulse wave velocity

In addition, we investigated the possible markers of vascular damage by determining the pulse wave velocity (PWV) (6). PWV was calculated as the distance/transit time (m/s) and was assessed by measuring the carotid aortic pulse wave (16).

### 2.8 Statistical analysis

The continuous variables and clinical and echocardiographic data are displayed as mean ± standard deviation, if normally distributed, or as median and interquartile range (IQR), if not normally distributed. The normality distribution was tested using the Shapiro–Wilk test. The binomial and ordinal qualitative variables are presented as frequencies and percentages. The correlation between the continuous variables was tested using Pearson's correlation or Spearman's test. The comparison between the two groups was assessed using the Student’s *t*-test or Mann–Whitney test. The comparison between multiple groups was assessed by one-way ANOVA or the Kruskall–Wallis test. A two-sided *p*-value of 0.05 was considered statistically significant for all tests. All statistical analyses were performed with SPSS software version 25.0 (International Business Machines Corporation, Armonk, NY, USA).

## 3 Results

### 3.1 Population characteristics

Of the 38 initially eligible patients, 7 were excluded due to refusal to participate in the study. Overall, we studied 31 patients, of whom 21 were male (67.7%) and 10 were female (32.3%). Patients’ diabetic features are shown in Table 1.

**Table 1:** Clinical features of diabetic patients and healthy volunteers.

	Diabetic patients (*n* = 31)	Healthy volunteers (*n* = 31)	*p*-value
Age (years)	16.4 ± 3.1	15.8 ± 1.3	0.134
Weight (kg)	66.5 ± 12.2	61.8 ± 11.9	0.131
Height (cm)	170.7 ± 8.8	168 ± 9.1	0.239
BSA (m^2^)	1.8 ± 0.2	1.7 ± 0.2	**0**.**037**
BMI (kg/m^2^)	22.7 ± 3	21.8 ± 3.3	0.292
Systolic blood pressure (mmHg)	120 ± 9.3	116 ± 8	0.068
Diastolic blood pressure (mmHg)	75 ± 6.1	70 ± 5	**0**.**001**

Values are reported as mean ± SD, and *p*-values were calculated using Student’s *t*-test.

*p*-values in bold indicate statistically significant relationships.

BSA, body surface area; BMI, body mass index.

All patients presented an advanced pubertal stage (at least G3, PH3, and I3 for males and B4, PH4, and I3 for females). Average BMI fell into the normal range (around 75th percentile). Nine patients (29%) had a BMI score in the overweight range (BMI between the 85th and 95th percentile), while one patient had a BMI above the 95th percentile. There were no underweight patients.

Considering blood pressure values, four patients (12.9%) had both systolic and diastolic hypertension, while two patients had isolated diastolic hypertension (6.5%), as shown in Table 2. Six patients showed pressure values between the 90th and 95th percentile.

**Table 2:** Pressure percentiles for both systolic and diastolic pressure.

Percentiles	Systolic blood pressure	Diastolic blood pressure
<5°	1 (3.2%)	1 (3.2%)
5°–50°	4 (12.9%)	0
50°–90°	20 (64.5%)	20 (64.5%)
90°–95°	4 (12.9%)	6 (19.4%)
>95°	2 (6.5%)	4 (12.9%)

Thirty-one age- and sex-matched healthy volunteers enrolled in a previous study were also included. Their anthropometric features are reported in Table 1.

### 3.2 Glycemic control parameters

Regarding the time in range (TIR), six patients presented a value of >70% (19.4% of the population); 15 patients, between 50% and 70% (48.4% of the population); and 10 patients, <50% (32.3% of the population). Glycemic variability (CV) was <36% in 9 patients (29% of the population) and >36% in the remaining 21 patients (71% of the population). The test population had a median TAR of 24% (IQR 6) and a median TVAR of 12% (IQR 13). The HbA1c value of 12 patients at the last check-up was <7% (38.7%), and that of the remaining 19 was >7% (61.3%). If 6.5% is considered as the HbA1c cutoff value, 7 and 24 patients had a value below and above this threshold (22.5% and 77.5%, respectively).

### 3.3 Conventional echocardiographic parameters and glycaemic control

Overall, our population showed normal average values of diameter, volume, ejection fraction, cardiac mass, and diastolic function parameters. The average values and standard deviations or median values and interquartile ranges are reported in Table 3.

**Table 3:** Echocardiographic parameters of diabetic adolescents.

Echocardiographic parameters	Mean values ± standard deviation/median value (IQR)
Ejection fraction (%)	56.8 ± 6.7
LV EDVi (ml/m^2^)	55.5 ± 11.4
LV ESVi (ml/m^2^)	25.6 ± 6.4
Cardiac mass (g)	108.8 ± 35.6
Cardiac mass/BSA (g/m^2^)	60.6 ± 15.3
Cardiac mass/height^^2.7^ (g/cm^^2.7^)	25.3 ± 6.5
LA diameter (cm)	2.8 ± 0.4
LA volume (ml)	37 ± 12.3
LA reservoir strain (%)	42.3 ± 9.5
*E*/*A*	1.7 ± 0.4
DT (ms)	178.6 ± 34.9
*E*/*e*′	5.3 ± 0.7
Septal *E*′ (cm/s)	14 ± 2
Lateral *E*′ (cm/s)	19 (7)
GLS (%)	−16.7 ± 2.2
Circumferential strain (CS, %)	−22.5 ± 3.4

Values are reported as mean ± SD or median (IQR).

LV EDVi, left ventricle end-diastolic volume indexed for body surface; LV ESVi, left ventricle end-systolic volume indexed for body surface; BSA, body surface area; LA, left atrium; *E*/*A* = early-to-late inflow velocity; DT, deceleration time, ratio; *E*/*e*′, inflow to relaxation velocity ratio; GLS, global longitudinal strain; PWV, pulse wave velocity.

The analysis of our population revealed a statistically significant trend toward higher septal thickness in patients with lower time in range (7.6 ± 1.7 mm in patients with TIR < 50% vs. 6 ± 1.2 mm in patients with TIR > 70%, *p* = 0.004). In addition, the diastolic parameters showed a correlation with glycemic control. *E*′ lateral velocity, in fact, appeared to be lower in patients with lower time in range (18 IQR 0.6 cm/s vs. 22 IQR 0.4 cm/s, *p* = 0.013). The same trend was also observed for septal *E*′ (13.8 ± 0.2 cm/s vs. 16.5 ± 0.1 cm/s, *p* = 0.01). When considering *E*′ velocities and other parameters of glycemic controls, the same relation was observed: *E*′ septal was lower for control variability >36% (*p* = 0.03), *E*′ lateral was lower in the group with glycated hemoglobin >6.5% (*p* = 0.01), and *E*′ septal negatively correlates with the total time above range (TTAR) (*R*^2^ = 0.201, *p* = 0.022). Moreover, the left atrium diameter was also higher in patients with less glycemic control (2.9 ± 0.3 cm vs. 2.4 ± 0.5 cm, *p* = 0.011) and positively correlated with TTAR (*R*^2^ = 0.258, *p* = 0.003).

### 3.4 Speckle tracking echocardiography and myocardial work

Regarding speckle tracking-derived parameters, we did not find any statistically significant relation with glycemic control. However, GLS showed a trend toward more positive values in patients with lower time in range (GLS −17.1% ± 2.3 in patients with TIR > 70%, while GLS −15.8% ± 1.6 in patients with TIR <50%, *p* = 0.346). Circumferential strain (CS), instead, showed higher values in patients with worst glycemic control: CS −21.9% ± 3.5 in patients with CV < 36% vs. CS −22.7% ± 3.4 in patients with CV >36%; CS −21.7% ± 2.8 in patients with glycated hemoglobin <6.5% vs. CS −22.7% ± 3.5 in patients with glycate hemoglobin >6.5%; and CS −21.5% ± 3 in patients with TIR > 70% vs. CS −22.9% ± 4.1 in patients with TIR < 50%. These relations did not reach statistical significance but highlighted possible trends.

GLS showed a good negative correlation with GCW (*r* = −0.708, *p* < 0.001), GWI (*r* = −0.572, *p* = 0.013), and GWE (*r* = −0.484, *p* = 0.006). No correlation was found between GLS and GWW. Finally, GLS was found to correlate with septal thickness, showing more positive values for increased thickness (*r* = 0.344, *p* = 0.016).

Similarly, GWW appeared to correlate with cardiac mass (*ρ* = 0.361, *p* = 0.046) and septal width during diastole (*ρ* = 0.381, *p* = 0.034).

### 3.5 Pulse wave velocity

**Figure 1:**
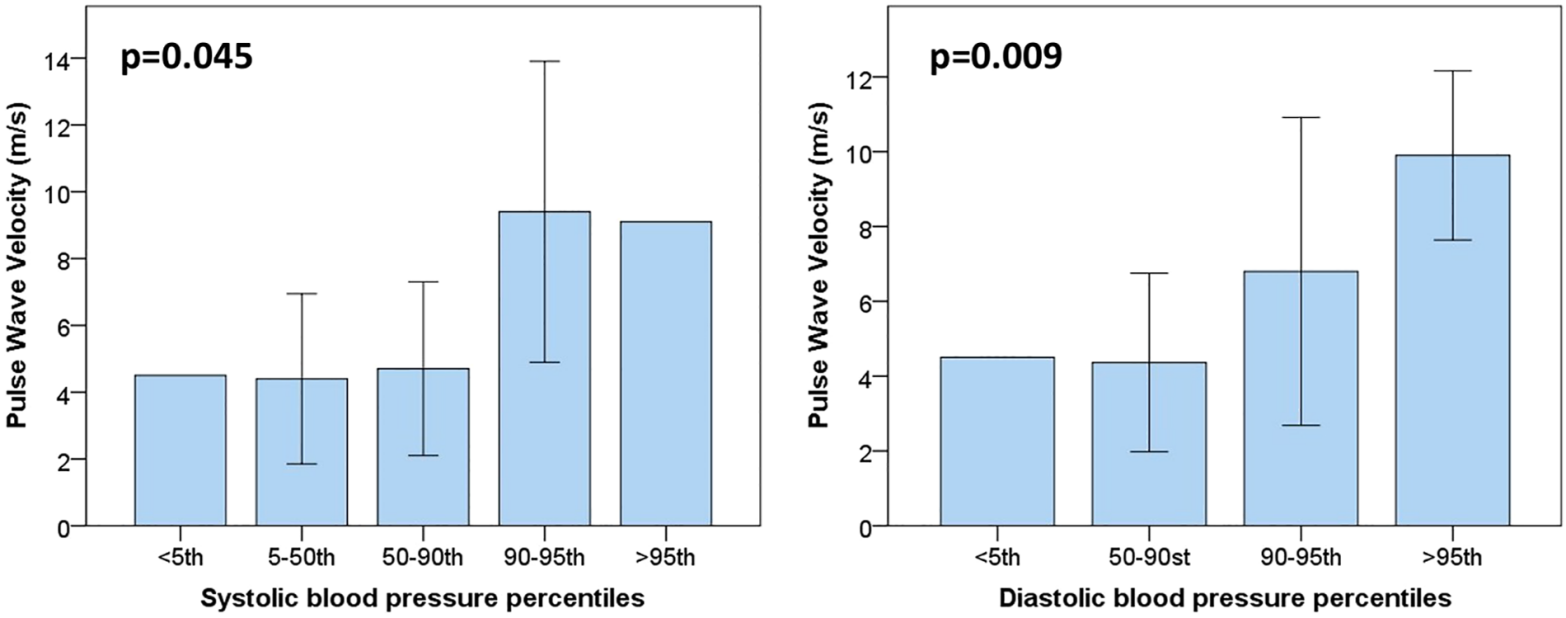
Pulse wave velocity values are plotted for systolic (on the left) and diastolic blood pressure percentiles (on the right). As it is possible to notice, PWV values start to be altered from pressure values above the 90th percentile (pre-hypertension state).

The median pulse wave velocity (PWV) in our population was around 5.2 m/s (2.7). As shown in Figure 1, there was a positive association between PWV with hypertension or pre-hypertensive state, both for systolic and diastolic blood pressure (*p* = 0.045 among different percentiles of systolic blood pressure; *p* = 0.009 for different percentiles of diastolic pressure). In patients with a pre-hypertensive state or first-degree hypertension, PWV reached pathological values around 8–10 m/s. However, no correlation was found with glycemic control, GLS, and the descriptive parameters of myocardial work.

### 3.6 Comparison with healthy volunteers

**Figure 2:**
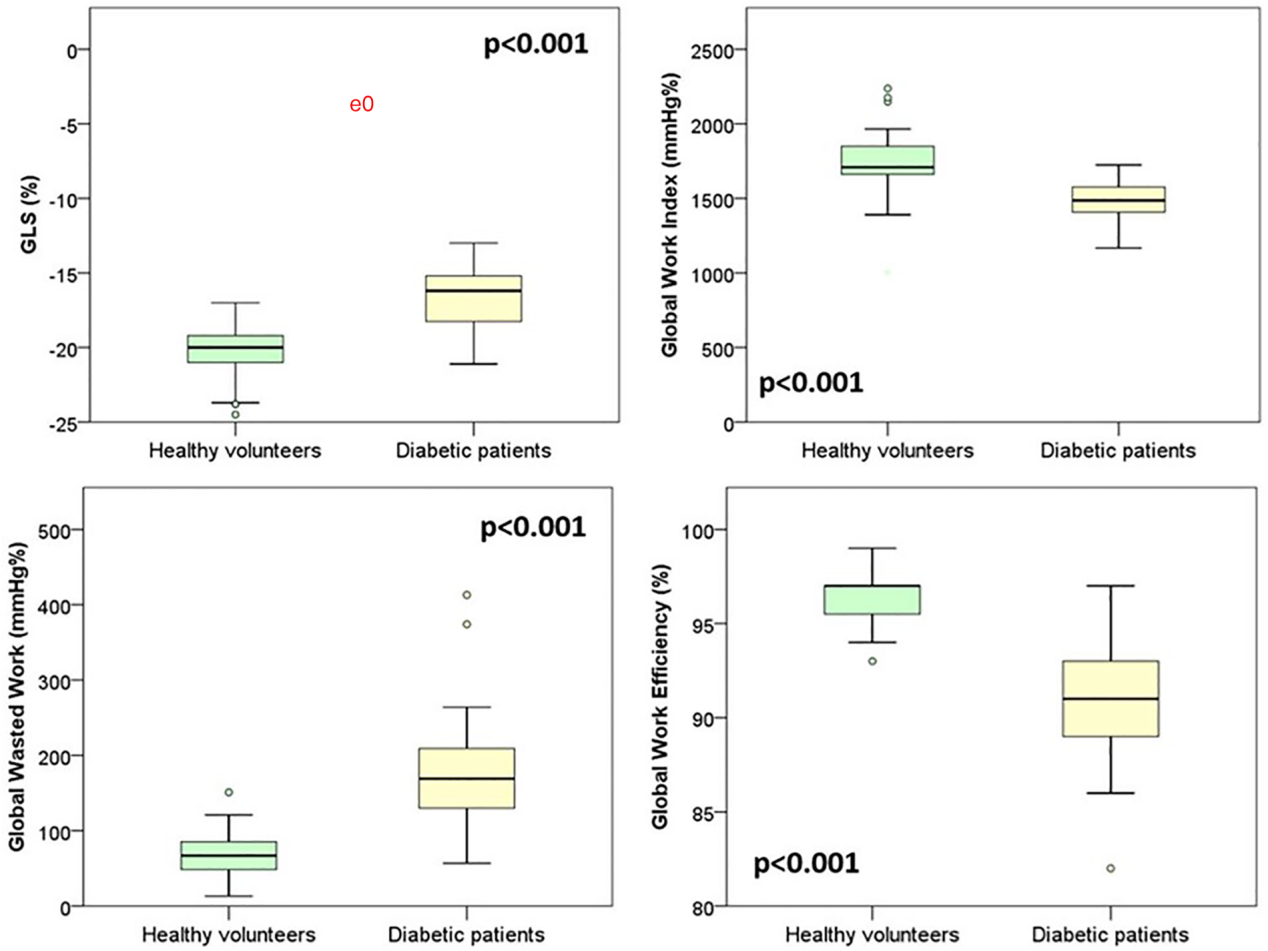
Comparison of speckle tracking and myocardial work data between diabetic patients and healthy volunteers.

GLS and myocardial work data from diabetic adolescents were also compared with the data from 31 age- and sex-matched healthy volunteers from a previous study in our lab (Table 4). Although ejection fraction did not show any impairment, GLS and myocardial work indexes appeared to be significantly impaired if compared to those of our reference population. Diabetic patients showed significantly lower values of GLS (−16.7% ± 2.2 in diabetic patients vs. −20.5% ± 1.9 in healthy volunteers, *p* < 0.001). As shown in Figure 2, the myocardial work parameters appeared to be significantly different in diabetic patients with lower global work index (1,466 mmHg% ± 148 in diabetic patients vs. 1,744 ± 234 mmHg% in healthy volunteers, *p* < 0.001), increased global wasted work (169 mmHg% IQR 86 in diabetic patients vs. 67 mmHg% IQR 39 in healthy volunteers, *p* < 0.001), and lower global work efficiency (91% IQR 4 in diabetic patients vs. 96.8 IQR 6 in healthy volunteers, *p* < 0.001). No significative difference was found among the amount of global constructive work [1,941 (491) mmHg% in diabetic adolescents vs. 2,120 (280) mmHg% in healthy volunteers, *p* = 0.097].

**Table 4:** Speckle tracking and myocardial work data in diabetic patients and healthy volunteers.

	Diabetic patients *n* = 31	Healthy controls *n* = 31	*p*-value
GLS (%)	−16.7 ± 2.2	−20.5 ± 1.9	**<0.001**
GWI (mmHg%)	1,466 ± 148	1,744 ± 234	**<0.001**
GCW (mmHg%)	1,941 (491)	2,120 (280)	0.097
GWW (mmHg%)	169 (86)	67 (39)	**<0.001**
GWE (%)	91 (4)	96.8 (6)	**<0.001**

Values are reported as mean ± SD or median (IQR) based on the numerical distribution of variables. *p*-values were calculated using the Student’s *t*-test or the Mann–Whitney test.

*P*-values in bold indicate statistically significant relationships.

GLS, global longitudinal strain; GWI, global work index; GCW, global constructive work; GWW, global wasted work; GWE, global work efficiency.

## 4 Discussion

This paper presents the data from a cross-sectional study on non-invasive conventional and advanced echocardiographic assessment in adolescents diagnosed with type 1 diabetes mellitus for at least 10 years. We found that GLS, global work index, global wasted work, and global work efficiency were impaired in our population if compared to healthy volunteers. Moreover, conventional echocardiography showed a significative trend toward early impairment of diastolic parameters in patients with the worst glycemic control, and pulse wave velocity fell into the pathological range for hypertensive and pre-hypertensive patients.

To the best of our knowledge, this appears to be the first study to explore the existence of early cardiovascular complication markers using advanced echocardiography techniques (MW) in a population of adolescents with type 1 diabetes mellitus.

Regarding glycemic control assessment, our sample consistently fell outside the target values in the ISPAD guidelines indicative of good control and protection against the long-term risk of complications, for all examined parameters [HbA1c, time in range (TIR), time above range (TAR), time below range, coefficient of variation (CV)] (17, 18). Overall, the majority of subjects exhibited high glycemic variability, with a low TIR, reflected in HbA1c levels that averaged above the desirable target in specialized and technologically advanced settings (6.5%). This suggests a prolonged influence of this risk factor in most subjects.

Moreover, our sample showed also suboptimal pressure control. Comparing systolic and diastolic values with pediatric reference tables for age and height, 12 out of 31 patients (38.7%) showed significant blood pressure alterations: 6 were in the “elevated pressure” range (systolic or diastolic blood pressure >90th percentile but <95th percentile), while the other 6 had stage 1 hypertension (19). These data confirm the potential utility of afterload-independent cardiac function parameters.

Speckle tracking data from diabetic adolescents were compared with the data from 31 age- and sex-matched healthy volunteers enrolled in a previous study. Two samples were comparable in terms of age, weight, and height, whereas the diabetic adolescents seemed to have significantly higher BSA (*p* = 0.037) and increased diastolic pressures (*p* < 0.001) if compared to the healthy subjects. Nevertheless, since the literature suggests the independence of global longitudinal strain (GLS) from BSA, a comparison of the two samples was deemed feasible. The comparison revealed significantly lower (more positive) GLS in the diabetic adolescents cohort (−16.7% ± 2.2 in diabetic patients vs. −20.5% ± 1.9 in healthy volunteers, *p* < 0.001). Further investigation into myocardial work showed a significantly lower global work index (*p* < 0.001) and global work efficiency (*p* < 0.001) along with a higher global wasted work (*p* < 0.001), as if there was an increased energy loss and futile work during both contraction and isovolumetric relaxation. Surprisingly, GCW resulted comparable between diabetic patients and healthy volunteers. These data suggest that cardiac mechanical impairment is more attributable to a rise in futile work during systole and isovolumetric relaxation than to a loss of positive work in the same phases. Whether this pattern of subclinical dysfunction might be linked to the specific damages related to hyperglycemia remains to be understood.

The existing literature on myocardial work and adult diabetic patients is mostly focused on type 2 diabetes mellitus. In a recent study on type 2 diabetic adult patients, Liao et al. (20) observed decreased GLS, GWI, and GWE and increased GWW. Similar to our study, global constructive work was comparable between patients and controls. These changes observed in GLS and MW descriptive parameters are reversible by improving glycemic control (21).

These parameters significantly reflect myocardial involvement, and we therefore suggest their integration into the clinical follow-up of these patients.

Given the extensive literature documenting the predictive power of GLS for unfavorable long-term cardiovascular outcomes, our finding of reduced GLS among diabetic patients should raise particular concern since multiple risk factors may coincide during growth and adulthood in this patient category. Although, as previously mentioned, our sample did not exhibit statistically significant correlations or associations between GLS, global circumferential strain (GCS), and glycemic control parameters, a trend was observed based on our measurements: more negative GLS values in individuals with better glycemic control parameters and, conversely, more negative GCS values in individuals with worse glycemic control. This observation, consistent with the literature findings for other pathologies causing primary GLS reduction, may be attributed to an initial compensatory mechanism by GCS, which would be consistently increased in individuals with worse GLS (5). However, the validity of this explanatory hypothesis in the context of our sample remains to be demonstrated (22–27). The lack of correlation between speckle tracking data and glycemic control parameters in our opinion can be explained by the substantial homogeneity of our sample in terms of glycated hemoglobin, time in range, and coefficient of variation.

In line with the available literature on left ventricular hypertrophy with unfavorable cardiovascular outcomes and the onset of ventricular arrhythmias, GLS and GWW in our study exhibited a positive correlation with interventricular septum thickness (less negative GLS values for greater interventricular septum thickness; increased wasted work for higher septal thickness) (28, 29).

In literature, left ventricular thickness and cardiac mass are reported to show an early tendency to increase in diabetic patients. However, in our sample, cardiac mass did not differ significantly from that of healthy subjects from previous studies. This might suggest that subclinical alterations in myocardial function appear in the natural history of type 1 diabetes mellitus before actual hypertrophic adaptation occurs.

Concerning diastolic functionality parameters, no frank pathological alteration was evident in the echocardiographic parameters of individuals in our sample. However, TDI diastolic function parameters and left atrial diameters showed a deteriorating trend with worse glycemic control. Both septal and lateral *E*′ velocities were higher in individuals with TIR > 70% compared to those with lower TIR; lateral *E*′ velocity was significantly higher in individuals with onset HbA1c below 6.5%, and septal *E*′ velocity was significantly higher in individuals with CV ≤36%. Additionally, larger left atrial diameters were observed in individuals with HbA1c above 6.5%. A statistically significant correlation was found between left atrial diameter and TVAR and between septal *E*′ velocity and TVAR. These data align with the existing literature indicating that one of the earlier cardiovascular alterations in type 1 diabetes mellitus involves diastolic function parameters before ejection fraction impairment. The correlation between TVAR and these parameters is also explained by preclinical studies on the effects of hyperglycemia and its variations on tissues, demonstrating how even short-term and non-sustained glycemic alterations can be responsible for subclinical cardiac dysfunction (30–32). On the other hand, some studies in the literature show that even relatively short periods (e.g., 1 month) of improved glycemic control can lead to a significant improvement in cardiac performance (33).

Finally, this study also considered indices of vascular damage. Pulse wave velocity (PWV) is a sensitive marker of arterial stiffness and consequently cardiovascular outcomes. As in previous studies, our data showed no correlation between PWV and glycemic control parameters (34). On the other hand, the positive association between PWV and blood pressure values was confirmed (35–37). Comparing our data to the reference values in the literature, we found that PWV from our diabetic adolescents fell on average in between the 50th and 75th percentile (38). However, in patients with a pre-hypertensive state or first-degree hypertension, PWV fell into the pathological range reaching values superior to 8 m/s (>99th percentile of PWV) (38). These data suggest the necessity to raise our awareness toward hypertension and maximize our efforts to effectively screen our diabetic adolescents.

Although clinical manifestations occur in adulthood, atherosclerosis is a continuum process that begins early in childhood, especially if major risk factors, such as diabetes and hypertension, are present. Thus, it is particularly important to address cardiovascular risk factors early in life as clearly emphasized in the clinical practice guidelines. On the same note, there is a need for further research to fully understand the impact of T1D on the heart and blood vessels of adolescents. These investigations can contribute to the development of targeted preventive and therapeutic strategies to preserve cardiovascular health in this population of diabetic patients.

## 5 Limitations

This study has some limitations. The small number of individuals in our sample is the most relevant, as statistical analyses may not have highlighted results suitable for a comprehensive discussion due to the insufficient number of subjects. However, our patients represent a very well-selected cohort since the patients were diagnosed with diabetes for at least 10 years and showed comparable anthropometric features and glycemic control parameters. Moreover, the patients were scanned under the same conditions over a short amount of time.

## 6 Conclusions

Conventional echocardiography showed a significative trend toward early impairment of diastolic parameters in patients with the worst glycaemic control. Moreover, both global longitudinal strain and descriptive parameters of myocardial work were significantly altered in the sample examined compared to healthy individuals, highlighting a subclinical cardiac damage already present during adolescence. The analysis of pulse wave velocity showed pathologically increased arterial stiffness in diabetic patients with blood pressure above the 90th percentile. Therefore, PWV, GLS, and myocardial work can be useful tools in assessing cardiovascular function in diabetic patients, while preventive strategies should be implemented to maximize glycemic and pressure control effectiveness.

Additional investigations, with long-term follow-up and large-scale cohorts, will be necessary to correlate myocardial strain, myocardial work alteration, and pulse wave velocity with the clinical outcomes of this patient group.

## Data Availability

The raw data supporting the conclusions of this article will be made available by the authors, without undue reservation.

## Supplementary material

Supplementary Figure 1 A summary outline of the study design and its main results are reported. DM1, type 1 Diabetes; HbA1, glycated haemoglobin; TIR, time in range; TAR, time above range; TVAR, time very above range; GLS, global longitudinal strain; PWV pulse wave velocity; GWI, global work index; GCW, global constructive work; GWW, global wasted work; GWE, global work efficiency; E/e', inflow to relaxation velocity ratio.
