# Supplementary figures and images for: Cardiovascular function shows early impairment in asymptomatic adolescents diagnosed with type 1 diabetes mellitus: an ultrasound-derived myocardial work study
Source: Front Cardiovasc Med. 2025 Feb 5;11:1476456. doi: 10.3389/fcvm.2024.1476456 (PMC11835514; doi:10.3389/fcvm.2024.1476456)

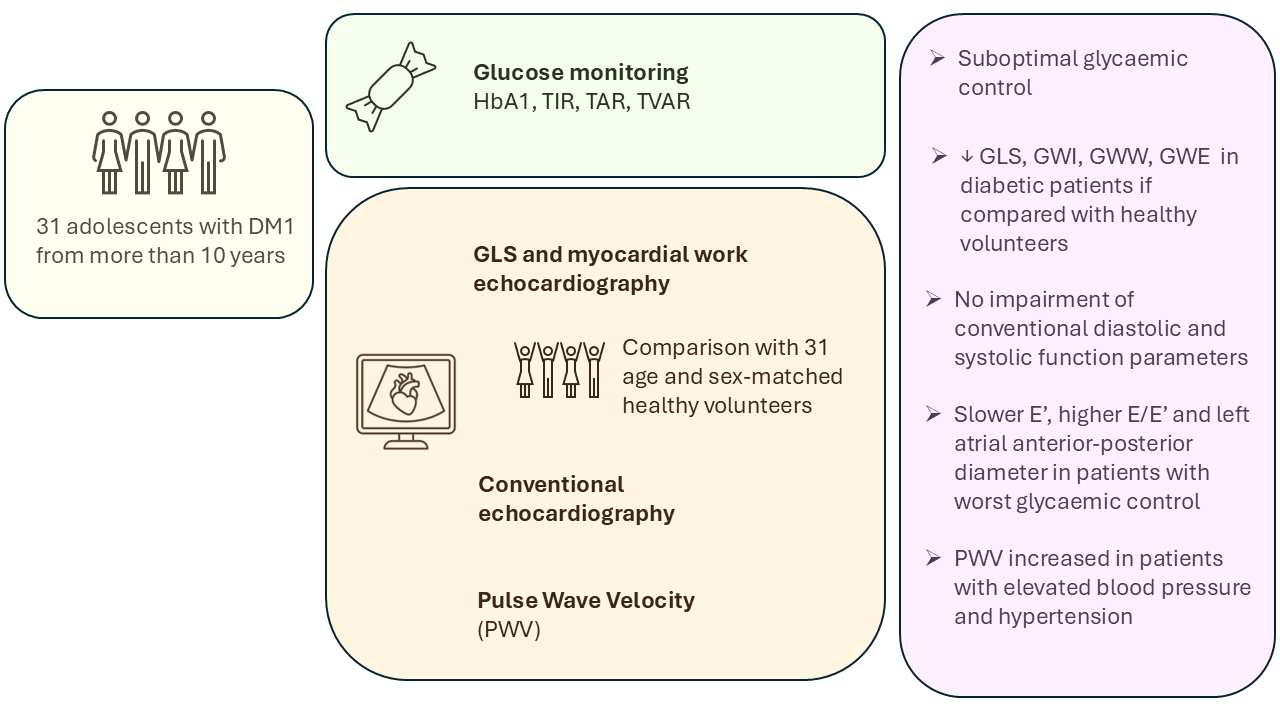

Supplement: Supplementary Figure 1 — A summary outline of the study design and its main results are reported. DM1, type 1 Diabetes; HbA1, glycated haemoglobin; TIR, time in range; TAR, time above range; TVAR, time very above range; GLS, global longitudinal strain; PWV pulse wave velocity; GWI, global work index; GCW, global constructive work; GWW, global wasted work; GWE, global work efficiency; E/e', inflow to relaxation velocity ratio. [file Image1.tif]
